# Association of Hemoglobin Level and Diagnostic Delay with Left Atrial Volume Index in Patients with Familial Mediterranean Fever: A Cross-Sectional Observational Study

**DOI:** 10.3390/jcm15197491

**Published:** 2026-09-26

**Authors:** Bayram Kızılkaya, Osman Cüre, Hüseyin Durak, Mustafa Çetin, Mehmet Serhat Topaloğlu

**Affiliations:** 1 Department of Internal Medicine, Recep Tayyip Erdoğan University, 53100 Rize, Türkiye; 2 Department of Rheumatology, Faculty of Medicine, Recep Tayyip Erdoğan University, 53100 Rize, Türkiye; 3 Department of Cardiology, Faculty of Medicine, Recep Tayyip Erdoğan University, 53100 Rize, Türkiye; 4 Department of Physical Medicine and Rehabilitation, Recep Tayyip Erdoğan University, 53100 Rize, Türkiye

**Keywords:** familial mediterranean fever, left atrial volume index, hemoglobin, diagnostic delay, echocardiography

## Abstract

**Background/Objectives:** Subclinical cardiac involvement may occur in familial Mediterranean fever, even outside acute attacks. However, the factors associated with variation in left atrial volume index (LAVI), particularly the roles of hemoglobin levels and diagnostic delay, remain poorly understood. This study aimed to evaluate the association between hemoglobin levels, diagnostic delay, and LAVI in patients with FMF. **Methods:** This cross-sectional observational study included 98 FMF patients with preserved left ventricular ejection fraction (LVEF ≥ 50%). Demographic, clinical, laboratory, and echocardiographic parameters were assessed. The relationships between LAVI, hemoglobin levels, and diagnostic delay were investigated using correlation analyses and multivariable linear regression. **Results:** Patients were 35.3 ± 12.4 years old on average, and 45.9% were men. The mean hemoglobin level, diagnostic delay, and LAVI were 13.51 ± 1.63 g/dL, 8.90 ± 9.94 years, and 18.32 ± 4.67 mL/m^2^, respectively. LAVI was inversely correlated with hemoglobin (r = −0.454, *p* < 0.001) and positively correlated with diagnostic delay (r = 0.262, *p* = 0.009). In the final multivariable model, hemoglobin (β = −0.333, *p* < 0.001), LVEF (β = 0.244, *p* = 0.006), and LVMI (β = 0.265, *p* = 0.002) were independently associated with LAVI, whereas diagnostic delay (β = 0.212, *p* = 0.017) and lateral a′ (β = 0.215, *p* = 0.014) were considered exploratory associations. **Conclusions:** In FMF patients, lower hemoglobin levels were independently associated with higher LAVI values, whereas diagnostic delay showed an exploratory association. These findings are hypothesis-generating and require confirmation in longitudinal studies.

## 1. Introduction

Familial Mediterranean Fever (FMF) is a hereditary autoinflammatory disorder in which inflammatory activity may persist even when patients are clinically free of attacks. Such ongoing inflammation may gradually affect vascular integrity, favor atherosclerotic changes, and thereby contribute to an increased long-term cardiovascular burden [1]. Although cardiac involvement in FMF has classically been associated with pericarditis and amyloidosis, recent years have shown that inflammation can directly affect cardiac structure and function [2]. Subclinical cardiac involvement warrants particular attention, as conventional and tissue Doppler echocardiography may reveal early functional abnormalities despite the absence of clinically apparent attacks [3].

Nevertheless, previous investigations have largely emphasized ventricular function, left atrial size and function, particularly in individuals with preserved left ventricular systolic function. Left atrial volume index (LAVI) is considered a sensitive indicator of chronic diastolic burden and long-term cardiovascular risk and has been shown to be associated with systemic inflammation [4,5,6]. Lower hemoglobin levels have also been associated with cardiac remodeling, heart failure, and increased filling pressures [7,8,9].

Diagnostic delay is common in FMF, and long diagnostic delays increase the inflammatory burden, leading to organ damage and are associated with the development of AA amyloidosis [10,11]. However, the effect of diagnostic delay on variation in LAVI has not yet been clearly established. Furthermore, comprehensive analyses evaluating hemoglobin level and diagnostic delay together with LAVI and other cardiac structural parameters in the same model are limited [11,12,13].

We investigated whether hemoglobin level and diagnostic delay were related to left atrial volume index in patients with familial Mediterranean fever.

## 2. Methods

### 2.1. Study Design

This single-center, cross-sectional observational study enrolled 98 participants between April and June 2026 from the Rheumatology and Cardiology Departments of a tertiary research hospital in Rize Province, Türkiye. Approval of the study protocol was obtained from the Ethics Committee of Recep Tayyip Erdoğan University (24 April 2026; Decision No. 2026/204). The research was conducted in line with the Declaration of Helsinki, with written informed consent secured from all participants before their inclusion in the study.

### 2.2. Diagnosis of Familial Mediterranean Fever

The diagnosis of FMF was made based on the Tel-Hashomer criteria. Within the framework of these criteria, the main indicators include serositis involvement (peritonitis, synovitis or pleuritis) accompanied by recurrent fever attacks, unexplained AA type amyloidosis and a positive response to colchicine treatment. Secondary indicators are recurrent fever attacks, erysipelas-like skin rashes, and a history of FMF in first-degree relatives. It was considered sufficient for a patient to meet two main criteria or one main and two secondary criteria to receive an FMF diagnosis [14].

### 2.3. Eligibility Criteria

Eligible participants were adults with FMF (≥18 years) who had available echocardiographic records, complete clinical and laboratory data, and a history of consistent daily colchicine use. Exclusion criteria included a history of organ transplantation or malignancy, active or chronic infection, cardiomyopathy, rheumatic mitral valve disease, left ventricular ejection fraction (LVEF) < 50%, systemic inflammatory or hematological disease, use of medications affecting hemoglobin levels, iron deficiency anemia, abnormal vitamin B12 and folate levels, use of biological drugs, colchicine intolerance or non-compliance, and patients experiencing an FMF attack during the evaluation. For the purposes of this study, anemia was considered present when hemoglobin concentration was below 13.0 g/dL in men or below 12.0 g/dL in women, based on the 2024 World Health Organization criteria [15]. Patients with anemia related to chronic disease or inflammation were not excluded. Known iron deficiency anemia was excluded based on available clinical data. These criteria were established to minimize potential confounding factors that could affect the study results.

### 2.4. Clinical Information and Laboratory Assessment

Data collection covered demographic variables, including age, sex, and body mass index, as well as arterial blood pressure measurements. FMF-related clinical characteristics were recorded, including fever, abdominal pain, constipation, diarrhea, nausea, vomiting, chest pain, arthritis, arthralgia, erysipelas-like erythema, and the clinical response to colchicine. Age at symptom onset, age at diagnosis, duration of diagnostic delay, and the number of FMF attacks within the previous six months were also assessed. The evaluation further comprised physical examination findings, relevant comorbidities such as diabetes mellitus, hypertension, hyperlipidemia, and coronary artery disease, smoking and alcohol use, consanguinity, family history of FMF, and ongoing pharmacological treatment. Current medications were categorized as statins, angiotensin-converting enzyme inhibitors (ACE inhibitors), angiotensin II receptor blockers (ARBs), beta-blockers, diuretics, and antidiabetic agents. Information was collected through standardized participant questionnaires and corroborated using available medical records. The colchicine treatment dose (mg/day) was recorded from patient files and evaluated in the analyses.

Blood samples were obtained from participants in the morning following an overnight fasting period of at least 8 h. Laboratory assessment was conducted during a clinically inactive period, defined as a minimum of 2 weeks having elapsed since the latest FMF attack. The analyses comprised white blood cell and neutrophil counts, hemoglobin, fasting glucose, total cholesterol (TC), high-density lipoprotein cholesterol (HDL-C), low-density lipoprotein cholesterol (LDL-C), triglycerides (TG), C-reactive protein (CRP), fibrinogen, total protein, albumin, and creatinine. Estimated glomerular filtration rate (eGFR) was calculated to evaluate renal function using the Chronic Kidney Disease Epidemiology Collaboration (CKD-EPI) equation.

### 2.5. Disease Severity and Activity

FMF severity was quantified according to the International Severity Scoring System for FMF (ISSF). Based on the resulting score, disease was categorized as mild (0–2 points), moderate (3–5 points), or severe (≥6 points). Disease activity was determined with the Autoinflammatory Disease Activity Index (AIDAI), which incorporates 12 predefined symptoms recorded by patients in daily diaries over a 1-month period. Each symptom was coded as either present (1) or absent (0). An AIDAI score of ≥9 indicated active disease, whereas scores below 9 were interpreted as inactive disease [16,17].

### 2.6. Echocardiographic Evaluation

All participants underwent a comprehensive two-dimensional transthoracic echocardiographic examination performed by an experienced cardiologist. Standard 2D and M-mode recordings were used to obtain left atrial and left ventricular dimensions, including LV end-diastolic diameter (LVEDD) and LV end-systolic diameter (LVESD), together with pulsed- and continuous-wave Doppler parameters, mitral E- and A-wave velocities, and deceleration time (DT). Left ventricular ejection fraction (LVEF) was calculated using the modified Simpson method. At end-diastole, interventricular septal and posterior wall thicknesses were determined in the parasternal long-axis plane. Left ventricular mass (LVM) was calculated according to the Devereux equation, and the resulting value was indexed to body surface area (BSA) to obtain the left ventricular mass index (LVMI). BSA was calculated as 0.007184 × weight^0.425^ × height^0.725^. Left atrial volume was quantified with the area-length method using apical four-chamber (A4C) and two-chamber (A2C) views. Both maximal and minimal LA volumes were recorded, and the LAVI was calculated by indexing LA volume to BSA [18,19]. 

To assess the reproducibility of LAVI, the primary outcome, a random subset of 20 patients was re-analyzed. Intra-observer reproducibility was evaluated by the same observer re-measuring LAVI in a blinded manner after an interval of at least two weeks. Inter-observer reproducibility was assessed by a second experienced cardiologist who was blinded to the initial measurements. Agreement was quantified using the intraclass correlation coefficient (ICC; two-way random-effects model, absolute agreement) with 95% confidence intervals, and Bland–Altman analysis was used to estimate the mean bias and 95% limits of agreement.

### 2.7. Statistical Analysis

Data analysis was carried out primarily in IBM SPSS Statistics version 29 (IBM Corp., Armonk, NY, USA), while selected analyses were additionally undertaken in Python (version 3.14.6) with the Statsmodels and SciPy libraries. For continuous variables, normality was assessed using the Kolmogorov–Smirnov test and supported by visual inspection of histograms. Descriptive statistics were selected according to the observed distribution, with normally distributed variables expressed as mean ± standard deviation (SD) and non-normally distributed variables presented as median values with interquartile ranges (IQR). Categorical data are presented as frequencies and percentages. For comparisons between two independent groups, the independent-samples *t*-test was applied to normally distributed continuous variables, while the Mann–Whitney U test was used for variables that did not satisfy the normality assumption. Associations of LAVI with clinical and laboratory variables were first explored according to the distribution of the data. Pearson’s correlation was applied to normally distributed variables, whereas Spearman’s rank-based method was used when the normality assumption was not met. Variables demonstrating a significant univariable association with LAVI (*p* < 0.05) were subsequently entered into a multivariable linear regression analysis. Variable selection in the final model was performed through backward elimination. Multicollinearity among predictors was assessed using variance inflation factors (VIF). Model fit was evaluated using R^2^, adjusted R^2^, and the overall F-test. Residual normality was examined with the Jarque–Bera test and homoscedasticity with the Breusch–Pagan test. As heteroscedasticity was detected, heteroscedasticity-consistent (HC3) standard errors were additionally computed. The influence of individual observations was assessed using Cook’s distance (threshold 4/*n*), and the robustness of borderline associations was evaluated in sensitivity analyses excluding influential observations. Model estimates were presented as unstandardized regression coefficients (B), standardized β coefficients, 95% confidence intervals (CIs), and corresponding *p*-values. A two-sided *p*-value below 0.05 was considered indicative of statistical significance.

## 3. Results

### 3.1. Baseline Clinical, Laboratory, and Echocardiographic Characteristics

The study population comprised 98 individuals diagnosed with FMF. The mean age of the patients was 35.26 ± 12.37 years, and 45.9% of the participants were male. Left ventricular systolic function was preserved in the cohort (LVEF 60.22 ± 5.58%). The mean LAVI was found to be 18.32 ± 4.67 mL/m^2^. LVMI was 72.43 ± 15.75 g/m^2^. The mean lateral a′ velocity was 9.40 ± 2.49 cm/s. The corresponding values for hemoglobin, diagnostic delay, and disease duration were 13.51 ± 1.63 g/dL, 8.90 ± 9.94 years, and 14.03 ± 10.12 years, respectively (Table 1). Hemoglobin measurements were available for 96 of 98 patients. Among these patients, 12 (12.5%) met the criteria for anemia, including 3 of 45 men (6.7%) and 9 of 51 women (17.6%). The median hemoglobin concentration was 13.0 g/dL (IQR: 12.0–15.0), with a range of 9.9–17.0 g/dL. The mean hemoglobin concentration was 14.71 ± 1.29 g/dL in men and 12.46 ± 1.09 g/dL in women (Table 2).

LAVI measurements demonstrated excellent reproducibility. The intra-observer ICC was 0.96 (95% CI, 0.90–0.98) and the inter-observer ICC was 0.93 (95% CI, 0.83–0.97). Bland–Altman analysis revealed a mean bias of 0.3 mL/m^2^ (95% limits of agreement, −2.6 to +3.2 mL/m^2^) for intra-observer measurements and 0.7 mL/m^2^ (95% limits of agreement, −3.7 to +5.1 mL/m^2^) for inter-observer measurements, indicating no systematic measurement difference.

### 3.2. Relationship Between LAVI and Clinical and Laboratory Parameters

A moderate to statistically strong negative correlation was found between LAVI and hemoglobin levels (r = −0.454; *p* < 0.001). Patients with anemia had a significantly higher mean LAVI than those without anemia (22.56 ± 6.57 vs. 17.74 ± 3.66 mL/m^2^; Welch’s *t*-test, *p* = 0.028). Among the 12 anemic patients, hemoglobin was significantly and inversely correlated with LAVI (r = −0.692, *p* = 0.013). A weaker but significant inverse correlation was also observed among the 82 non-anemic patients with complete hemoglobin and LAVI data (r = −0.254, *p* = 0.021). Lower hemoglobin levels were associated with higher LAVI values (Figure 1). 

A significant and positive correlation was found between the time to diagnosis delay and LAVI (r = 0.262, *p* = 0.009) (Figure 2). A longer diagnostic delay was associated with higher LAVI. 

Additionally, LAVI was positively correlated with LVMI (r = 0.243; *p* = 0.017), LVEF (r = 0.207; *p* = 0.043), and disease duration (r = 0.224; *p* = 0.028), and negatively correlated with total protein level (r = −0.287; *p* = 0.005) (Table 1). These findings suggest that interindividual variation in LAVI may be associated with both cardiac and systemic factors.

### 3.3. Multivariable Linear Regression Analysis

Variables meeting the predefined significance threshold in the univariable analyses (*p* < 0.05) were entered into the initial multivariable linear regression model, followed by backward elimination. In the final ordinary least-squares (OLS) model, LAVI was associated with hemoglobin (standardized β = −0.333; *p* < 0.001), LVMI (standardized β = 0.265; *p* = 0.002), LVEF (standardized β = 0.244; *p* = 0.006), lateral a′ velocity (standardized β = 0.215; *p* = 0.014), and diagnostic delay (standardized β = 0.212; *p* = 0.017) (Table 3). Among these variables, hemoglobin showed the strongest association with LAVI, based on the absolute standardized β coefficient. The final model included 92 patients with complete data complete-case analysis; patients with missing values in any model variable were excluded, and the model explained 39.9% of the variance in LAVI (adjusted R^2^ = 0.364; F [5,86] = 11.409, *p* < 0.001). No substantial multicollinearity was observed: although diagnostic delay and disease duration were moderately correlated (r = 0.497), their VIFs in the initial model were 1.59 and 1.63, respectively, and VIFs for all predictors in the final model ranged from 1.02 to 1.11. Residual diagnostics indicated no marked departure from normality (Jarque–Bera *p* = 0.104), whereas the Breusch–Pagan test suggested heteroscedasticity (*p* = 0.003). Under HC3 inference, the associations of hemoglobin, LVMI, and LVEF with LAVI remained statistically significant, whereas those of diagnostic delay and lateral a′ did not reach the *p* < 0.05 threshold (diagnostic delay, *p* = 0.079; lateral a′, *p* = 0.073). Seven observations exceeded the conventional Cook’s distance threshold of 4/*n*, and the lateral a′ association was additionally sensitive to exclusion of the most influential observation. Accordingly, the associations of diagnostic delay and lateral a′ with LAVI should be regarded as exploratory rather than robust independent effects.

## 4. Discussion

In this study of FMF patients with preserved ejection fraction, hemoglobin showed the strongest association with LAVI and, together with LVMI and LVEF, remained significant under robust (HC3) inference, whereas diagnostic delay and lateral a′ were attenuated and are interpreted as exploratory. The clinical significance and underlying mechanisms of the hemoglobin association nonetheless remain uncertain.

Hemoglobin level may represent a systemic factor associated with variation in LAVI among patients with FMF. Lower hemoglobin levels may reduce myocardial oxygen delivery and promote compensatory hemodynamic responses that could influence cardiac chamber dimensions. Iron deficiency may promote structural alterations and fibrotic processes in the myocardium through oxidative stress, which could theoretically be associated with higher LAVI values [9,20]. However, iron status was not systematically assessed in our cohort; therefore, the contribution of iron deficiency to the observed association cannot be determined. Previous studies have reported higher LAVI and left atrial volume values in individuals with iron-deficiency anemia than in controls, with partial normalization of these measures following correction of anemia. Furthermore, chronic anemia has been associated with larger ventricular dimensions, higher LVMI, and elevated filling pressures, with reductions in these parameters after treatment. These observations suggest that persistent anemia may be associated with cardiac structural and hemodynamic adaptation involving both the ventricles and atria [9,21]. Overall, the available evidence supports an association between anemia and left atrial size, although the mechanisms underlying this relationship remain incompletely understood [22].

The positive association between diagnostic delay and LAVI raises the possibility that delayed diagnosis may serve as a marker of prolonged or insufficiently controlled inflammatory exposure. Diagnostic delay is common in FMF, and patients diagnosed later may experience longer periods of uncontrolled inflammation and a higher frequency of inflammation-related complications, including AA amyloidosis [23]. Subclinical inflammation has been associated with endothelial dysfunction and increased arterial stiffness, providing a potential biological context for the observed association between diagnostic delay and LAVI [24,25].

In the present study, both LVMI and LVEF were independently associated with LAVI, suggesting that variation in left atrial volume was related to ventricular structural and functional characteristics. Previous studies have associated higher LVMI and LAVI values with chronic volume loading and elevated diastolic filling pressures [26,27]. Higher left atrial volume values despite preserved LVEF have also been reported in association with diastolic dysfunction and chronic inflammation [28,29,30]. However, these mechanisms were not directly evaluated in our study, and the mean LAVI was 18.32 ± 4.67 mL/m^2^, well below the ASE/EACVI upper normal limit of 34 mL/m^2^. Therefore, our findings should be interpreted as associations with variation in LAVI around a group mean within the normal range rather than as evidence of pathological left atrial enlargement or remodeling.

Systemic factors, particularly hemoglobin levels and diagnostic delay, may be related to cardiac structural characteristics through inflammatory and non-inflammatory pathways. Previous studies have suggested that chronic inflammation and hematological abnormalities may influence atrial and ventricular structure through tissue hypoxia, oxidative stress, and fibrotic mechanisms [31,32]. In our study, lower hemoglobin levels and longer diagnostic delay were independently associated with higher LAVI values, although the group mean remained within the normal range. The clinical significance of these associations requires further investigation in longitudinal studies.

Study limitations: This study has certain limitations. The single-center setting and relatively small number of participants may restrict the extent to which our findings can be extrapolated to broader patient populations. Second, owing to the cross-sectional observational design, the associations of hemoglobin level and diagnostic delay with LAVI cannot be interpreted as causal or temporal relationships. Longitudinal changes in LAVI and their relationships with clinical outcomes could not be evaluated. Left atrial size was evaluated using LAVI alone; more sensitive assessments of left atrial structure and function, such as left atrial strain analysis, three-dimensional echocardiography, or cardiac magnetic resonance imaging, were not performed. Furthermore, the absence of measurements of IL-6, TNF-α, and other inflammatory cytokines limited our ability to investigate potential inflammatory mechanisms underlying the observed associations. Since MEFV mutation analyses were not available in all patients, the genotype-phenotype relationship could not be evaluated. MEFV genotypes may be associated with inflammatory burden, AA amyloidosis, and cardiac involvement; therefore, the absence of genotype data may have introduced residual confounding and limited the interpretation of the observed associations. The etiology of lower hemoglobin levels could not be systematically determined in all patients. Although known iron deficiency anemia and nutritional deficiencies were excluded based on available clinical data, iron deficiency or other nutritional causes could not be completely ruled out. Therefore, residual confounding related to the underlying cause of reduced hemoglobin may have influenced the observed association with LAVI. Finally, longitudinal follow-up was unavailable; therefore, it could not be determined whether variation in LAVI was associated with subsequent atrial fibrillation, heart failure, or other cardiovascular outcomes.

## 5. Conclusions

In patients with FMF, lower hemoglobin levels were robustly and independently associated with higher LAVI values, whereas diagnostic delay showed an exploratory association with LAVI. LVEF and LVMI were also independently associated with LAVI. However, mean LAVI remained within the normal range, so these findings should not be interpreted as evidence of pathological left atrial enlargement or remodeling. Given the cross-sectional design, these associations are hypothesis-generating and require confirmation in longitudinal studies.

## Figures and Tables

**Figure 1:**
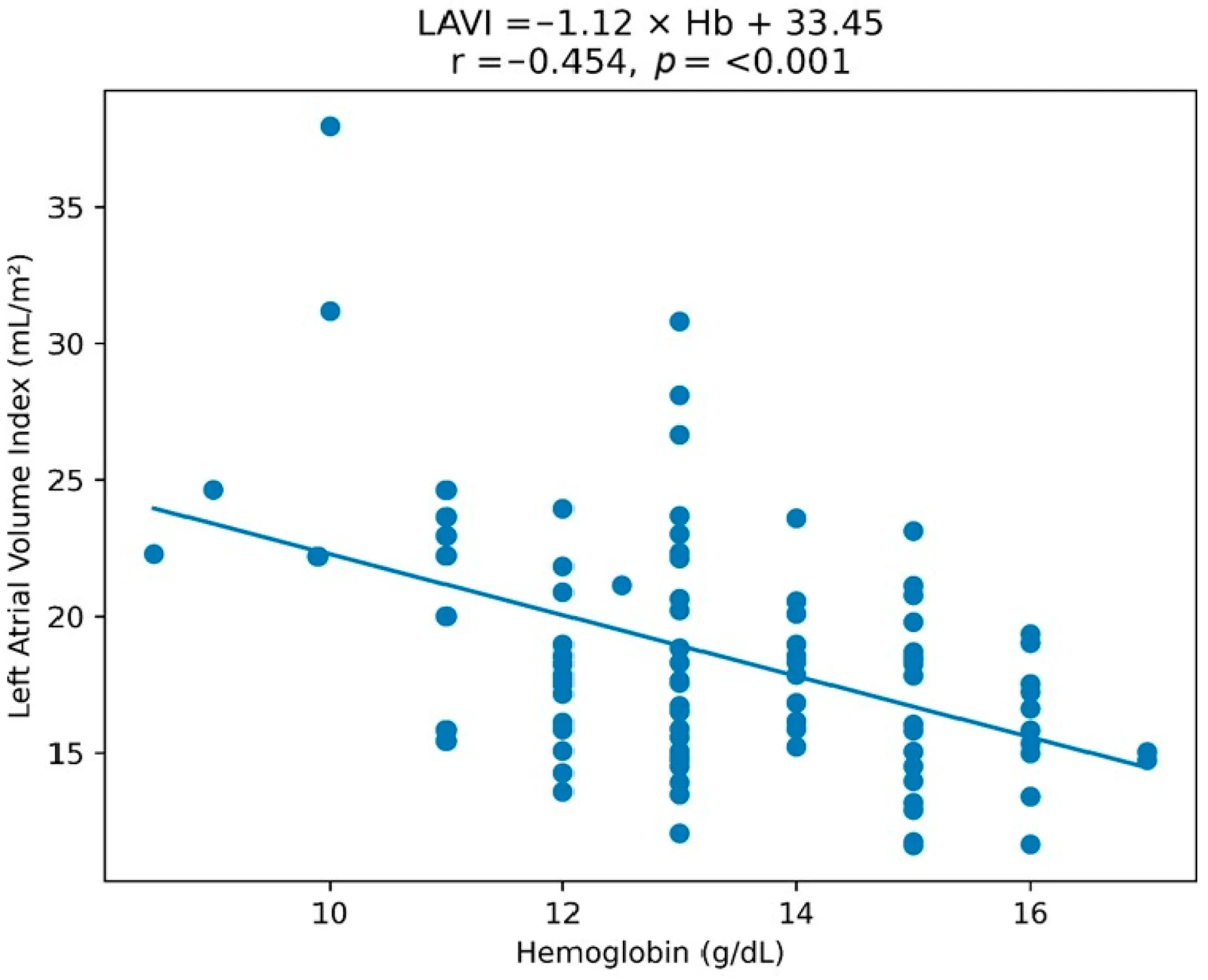
Relationship between hemoglobin (Hb) levels and left atrial volume index (LAVI) in patients with familial Mediterranean fever (FMF). The scatter plot demonstrates a significant inverse correlation between Hb levels and LAVI. Blue dots represent individual data points.

**Figure 2:**
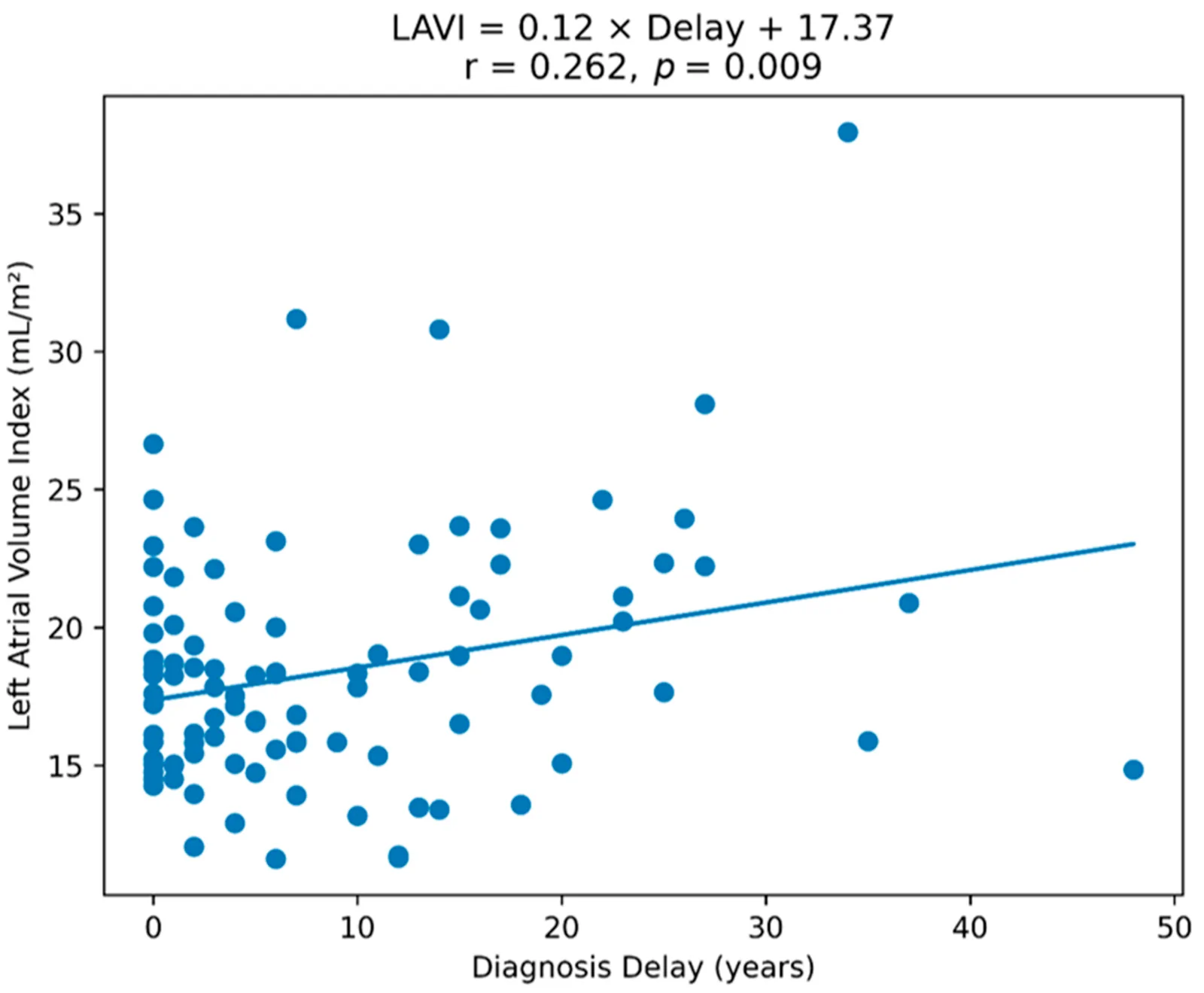
Relationship between diagnostic delay and left atrial volume index (LAVI) in patients with familial Mediterranean fever (FMF). The scatter plot demonstrates a significant positive correlation between diagnostic delay and LAVI. Blue dots represent individual data points.

**Table 1:** Baseline Clinical, Laboratory, and Echocardiographic Characteristics of Familial Mediterranean Fever Patients and Their Correlations with Left Atrial Volume Index (LAVI).

Variable	Baseline (All Patients)	r Value	*p* Value
Age (years)	35.26 ± 12.37	0.150	0.145
Sex (male), *n* (%)	45 (45.9%)	−0.311	0.002
BMI (kg/m^2^)	26.30 ± 4.92	−0.083	0.423
LVMI (g/m^2^)	72.43 ± 15.75	0.243	0.017
DM, *n* (%)	2 (2.0%)	−0.013	0.900
HPL, *n* (%)	37 (37.8%)	−0.027	0.797
HT, *n* (%)	6 (6.1%)	−0.060	0.561
Smoking, *n* (%)	20 (20.4%)	−0.028	0.789
Arthralgia, *n* (%)	70 (71.4%)	0.029	0.781
Arthritis, *n* (%)	10 (10.2%)	0.142	0.166
Chest pain, *n* (%)	58 (59.2%)	0.058	0.573
Chronic kidney failure, *n* (%)	1 (1.0%)	−0.019	0.856
Consanguinity, *n* (%)	31 (31.6%)	0.147	0.152
Constipation, *n* (%)	29 (29.6%)	0.072	0.487
Diarrhea, *n* (%)	26 (26.5%)	−0.065	0.531
FMF activity index	5.36 ± 1.77	−0.075	0.467
Diagnosis delay (years)	8.90 ± 9.94	0.262	0.009
FMF duration (years)	14.03 ± 10.12	0.224	0.028
First attack age (years)	16.57 ± 11.12	−0.159	0.122
FMF score	1.98 ± 0.35	0.194	0.058
Family history of FMF, *n* (%)	56 (57.1%)	0.085	0.410
Fever, *n* (%)	78 (79.6%)	0.103	0.320
Attacks (last 6 months, *n*)	2.24 ± 1.59	0.133	0.195
Muscle pain, *n* (%)	71 (72.4%)	0.101	0.326
Nausea, *n* (%)	68 (69.4%)	−0.107	0.298
Pleuritic pain, *n* (%)	46 (46.9%)	0.075	0.466
Skin rash, *n* (%)	9 (9.2%)	0.106	0.305
TSH mIU/L	2.30 ± 1.34	0.221	0.031
Albumin (g/dL)	4.54 ± 0.50	−0.115	0.266
CRP (mg/L)	5.00 [2.00–9.25]	−0.075	0.467
Creatinine (mg/dL)	0.75 ± 0.21	−0.068	0.510
FPG (mg/dL)	90.20 ± 9.33	−0.065	0.530
Fibrinogen (mg/dL)	346.81 ± 99.61	−0.115	0.272
HDL-C (mg/dL)	47.31 ± 10.41	0.091	0.379
Hb (g/dL)	13.51 ± 1.63	−0.454	<0.001
LDL-C (mg/dL)	114.34 ± 30.41	0.087	0.403
Neutrophils (10^3^/µL)	4.63 ± 2.00	0.009	0.932
TC (mg/dL)	186.89 ± 34.85	0.074	0.478
Total protein (g/dL)	7.58 ± 0.43	−0.287	0.005
TG (mg/dL)	124.87 ± 72.43	−0.089	0.392
WBC (10^3^/µL)	7.34 ± 2.04	0.018	0.860
eGFR (mL/min/1.73 m^2^)	108.62 ± 19.55	0.014	0.892
ACE/ARB, *n* (%)	4 (4.1%)	−0.085	0.410
CCB, *n* (%)	4 (4.1%)	−0.048	0.643
Colchicine dose (mg/day)	2.73 ± 0.62	0.217	0.033
LVEF (%)	60.22 ± 5.58	0.207	0.043
Mitral E (cm/s)	82.29 ± 19.43	−0.025	0.810
Mitral A (cm/s)	64.23 ± 16.84	0.125	0.224
E/A	1.30 ± 0.32	−0.192	0.062
Deceleration time (ms)	162.63 ± 50.99	−0.033	0.747
Lateral e′ (cm/s)	18.60 ± 3.9	−0.154	0.134
Lateral a′ (cm/s)	9.40 ± 2.49	0.260	0.011
E/e′	5.84 ± 1.98	0.071	0.490

Abbreviations: BMI: body mass index; LVEF: left ventricular ejection fraction; LVMI: left ventricular mass index; DM: diabetes mellitus; HPL: hyperlipidemia; HT: hypertension; TSH: thyroid-stimulating hormone; FMF: familial Mediterranean fever; CRP: C-reactive protein; FPG: fasting plasma glucose; HDL-C: high-density lipoprotein cholesterol; LDL-C: low-density lipoprotein cholesterol; TC: total cholesterol; TG: triglycerides; WBC: white blood cell count; eGFR: estimated glomerular filtration rate; ACE: angiotensin-converting enzyme; ARB: angiotensin II receptor blocker; CCB: calcium channel blocker; Hb: hemoglobin, E/A: ratio of early (E) to late (A) mitral inflow velocity, E/e′: ratio of early mitral inflow velocity (E) to early diastolic mitral annular velocity (e′).

**Table 2:** Distribution of Hemoglobin Levels According to Sex in Patients with Familial Mediterranean Fever.

Group	*n*	Mean ± SD	Median [IQR]	Min–Max (g/dL)
All patients	96	13.51 ± 1.63	13.0 [12.0–15.0]	9.9–17.0
Men	45	14.71 ± 1.29	15.0 [14.0–16.0]	11.0–17.0
Women	51	12.46 ± 1.09	13.0 [12.0–13.0]	9.9–15.0

Abbreviations: SD, standard deviation; IQR, interquartile range. Note: Hemoglobin data were available for 96 of 98 patients. Anemia was defined as hemoglobin < 13 g/dL in men and <12 g/dL in women.

**Table 3:** Factors Independently Associated with Left Atrial Volume Index (LAVI) in Patients with Familial Mediterranean Fever.

Variable	B	SE	Standardized β	%95 CI	t	*p*
Constant value	7.868	6.739	—	−5.528–21.264	1.168	0.246
LVEF	0.211	0.075	+0.244	0.062–0.361	2.809	0.006
LVMI	0.074	0.024	+0.265	0.027–0.121	3.143	0.002
Diagnostic delay	0.093	0.038	+0.212	0.017–0.170	2.430	0.017
Hemoglobin	−0.887	0.235	−0.333	−1.354–−0.420	−3.774	<0.001
Lateral a′	0.379	0.151	+0.215	0.080–0.678	2.517	0.014

Under HC3 inference, diagnostic delay and lateral a′ were no longer significant. Abbreviations: B, unstandardized regression coefficient; SE, standard error; β, standardized regression coefficient; CI, confidence interval; LVEF, left ventricular ejection fraction; LVMI, left ventricular mass index; Lateral a′, late-diastolic mitral annular velocity.

## Data Availability

The data presented in this study are available from the corresponding author upon reasonable request. The data are not publicly available due to privacy and ethical restrictions.
